# Baroreceptor reflex dysfunction in the BACHD mouse model of Huntington’s disease.

**DOI:** 10.1371/currents.RRN1266

**Published:** 2011-11-04

**Authors:** Analyne M. Schroeder, Dawn H Loh, Maria C. Jordan, Kenneth P. Roos, Christopher S. Colwell

**Affiliations:** ^*^Department of Psychiatry & Biobehavioral Sciences, Laboratory of Circadian and Sleep Medicine, University of California, Los Angeles, Los Angeles, CA, 90024 USA and ^‡^Department of Physiology, David Geffen School of Medicine at UCLA, Los Angeles, CA 90095-1751

## Abstract

Huntington’s disease is a progressive, neurodegenerative disorder that presents with a triad of clinical symptoms, which include movement abnormalities, emotional disturbance and cognitive impairment. Recent studies reported dysfunction of the autonomic nervous system in Huntington’s disease patients, which may contribute to the increased incidence of cardiovascular events in this patient population that often leads to death. We measured the baroreceptor reflex, a process dependent on proper autonomic function, in the BACHD mouse model of Huntington’s disease. We found a blunted response of the baroreceptor reflex as well as significantly higher daytime blood pressure in BACHD mice compared to WT controls, which are both indications of autonomic dysfunction. BACHD mice had increased heart weight to tibia length ratios at 7 and 12 mo of age suggesting hypertrophic changes of the heart, which we speculate is a response to the increased blood pressure and aberrant baroreceptor reflex. Despite these structural changes, the hearts of BACHD mice continue to function normally as assessed by echocardiographic analysis. Studies of autonomic and cardiovascular function in BACHD mice may help elucidate the pathophysiology of Huntington’s disease and aid in the development of clinical strategies to offset the incidence of fatal cardiovascular events in the Huntington’s disease patient population.

## Introduction

Huntington’s disease is an autosomal dominant neurodegenerative disorder characterized by apoptotic death of neurons in various brain regions that result in the progressive deterioration of movement, cognitive abilities and behavioral control [1].  In addition, Huntington’s disease patients are at increased risk for cardiovascular morbidity and are likely to succumb to cardiovascular events [2] [3].  Recent studies reported that individuals with Huntington’s disease display aberrant changes in the autonomic nervous system (ANS) that are detected even before the onset of other Huntington’s disease symptoms.  Misregulation of both the sympathetic and parasympathetic branches of the ANS have been described, whereby the sympathetic nervous system (SNS) becomes hyperactive in presymptomatic Huntington’s disease patients while the activity of the parasympathetic nervous system (PNS) progressively declines [4] [5] [6] [7] [8] [9].  These types of changes in the ANS are associated with poor prognosis for serious cardiovascular events that many times lead to death [10] [11] [12].  Further study of the cardiovascular system as well as autonomic activity in Huntington’s disease patients may help in better understanding disease pathology that may lead to clinical strategies to help curb the number of cardiovascular events in the patient population.

Many mouse models of Huntington’s disease have been created to recapitulate the various characteristics of the disease to allow for mechanistic investigations.  In some of the models, a cardiovascular phenotype has been documented [13] [14] [15] [16].  We are particularly interested in the BACHD model: a transgenic mouse that expresses the expanded form of the human *htt* gene that encode 97 stable glutamine repeats [17].  BACHD mice develop HTT aggregates similar to adult onset Huntington’s disease and display progressive motor deficits.  There is also evidence for circadian deficits along with indications that the ANS is affected as measured by heart rate variability [16].  Further investigations of this mouse model may help elucidate the autonomic and cardiovascular pathology in Huntington’s disease.

In this study, we investigated the cardiovascular phenotype in BACHD mice and report echocardiography, heart morphology and baroreceptor reflex properties in middle-aged mice.  The baroreceptor reflex is responsible for the overall tone as well as the acute moment-to-moment regulation of blood pressure (BP) by modulating cardiac output and total peripheral resistance [18] [19].  The central autonomic network, which includes the nucleus tractus solitarius (NTS) and other nuclei, receive and integrate BP information from baroreceptor cells located in the carotid artery and aortic arch.  The system responds by modulating autonomic outflow to various tissues of the body such as the heart, blood vessels and adrenal glands, in order to adjust BP accordingly [19].   Dysregulation of this reflex in the long run, may lead to detrimental consequences on the cardiovascular system [20].  Effects on the heart can be assessed using echocardiography and morphometry measurements, which look for changes in heart function and structure, respectively.  In our study, we find evidence for abnormal heart morphometry, changes in baseline BP, as well as a dysfunctional baroreceptor reflex response that would suggest dysregulation by the ANS.

## Methods

### Animals

BACHD mice on the C57BL6/J background [17] along with littermate wild-type (WT) controls were obtained from the mouse mutant resource at JAX (The Jackson Laboratory, Bar Harbor, Maine) in a colony maintained by the CHDI Foundation.  In order to obtain a sufficient number of animals for the baroreceptor studies, we included data from non-littermate (n=4/8) C57BL6/J WT mice.  Animals were housed in a controlled environment with a 12 hr light and 12 hr dark lighting cycle where lights-on occurred at 7am.  All the animals used in these studies were subject to all recommendations for animal use and welfare outlined by the UCLA Division of Laboratory Animals, as well as guidelines from the National Institutes of Health. The protocols for these studies were approved by the UCLA Animal Research Committee.

**

### Baseline Measurements and Baroreceptor Reflex

The administration of Angiotensin II (ATII) or Nitroprusside (NP) leads to episodes of hypertension or hypotension, respectively.  Changes in BP as a result of drug administration should elicit a compensatory response of heart rate (HR) via the baroreceptor reflex in order to normalize BP levels.  Differences in the ratio of change in HR to BP (∆HR/∆BP) may indicate aberrant signaling to the heart by the ANS.  Baroreceptor function was examined in WT (n=8) and BACHD (n=6) mice (7-9 mo of age) between the times of 11am (zeitgeber time, ZT 4) and 4pm (ZT 9).  During the experiment, HR was determined from the R-R interval of the electrocardiogram (EKG).  Mice were anesthetized and both femoral arteries were catheterized, whereby BP measurements were collected from one artery and drugs were administered through the other artery.  Following catheterization, isoflurane levels were decreased to between 1-1.5% and the mice were allowed to rest for at least 10 minutes.  Baseline BP and HR were then recorded.  Increasing concentrations of AT II (Low Dose (LD): 0.5, Mid Dose (MD): 1.0, High Dose (HD): 4.0 µg/kg) were administered, and each dose was followed by a flush of saline (+heparin 3U/mL) to ensure full delivery of the drugs.  Increasing concentrations of NP (LD: 10, MD: 20, HD: 40 µg/kg) were then administered similarly.  We spaced each drug infusion leaving at least 10-15 minutes between administrations to allow BP and HR to return to baseline levels.  As a control, we blocked both the muscarinic (75µg/kg glycopyrrolate) and β-adrenergic (750µg/kg propranolol) receptors before administering another HD dose of ATII and NP.  We calculated the absolute value of the maximum change in BP as well as the subsequent directional change in HR.  Ratios of these two values (∆HR/∆BP) reflect the magnitude HR change relative to the change in BP that characterizes the sensitivity of the baroreceptor response.

### Echocardiography and Heart Morphometry

In order to assess cardiac function, WT (n=6) and BACHD (n=5) mice (11-12 mo) were subject to a two-dimensional, M-mode echocardiography and spectral Doppler images acquired by a Siemens Acuson Sequoia C256 equipped with a 15L8 15MHz probe (Siemens Medical Solutions, Mountain View, CA) as previously described [21].  Mice were lightly anesthetized with 1% isoflurane vaporized in oxygen (Summit Anesthesia Solutions, Bend, OR).  The HR was determined from the R-R intervals of their EKG and was maintained at physiological levels (between 450 and 650 bpm) during the procedure.  Parameters measured include: Ventricular Septal Thickness (VST), End Diastolic Diameter (EDD), Posterior Wall Thickness (PWT), End Systolic Diameter (ESD), Aortic Ejection Time (Ao-ET), Left Ventricular percent Fractional Shortening (LV%FS), Left Ventricular Ejection Fraction (LvEF), Left Ventricular Mass (LVmass), and Early Diastole/Atrial contraction ratio (E/A). 

Body weight (BW), heart weight (HW) and tibia length (TL) were measured from 7-8 mo (WT: n=5; BACHD: n=7) and 12 mo old (WT: n=6; BACHD: n=5) mice.  HW/TL or HW/BW ratios were calculated to determine if there are differences in gross heart morphometry.

### Statistics

Baseline BP, HR as well as echocardiogram parameters were compared using a Student’s t-test.  A two-way repeated measures ANOVA was used to analyze the Baroreceptor data (genotype x drug concentration) and a two-way ANOVA was used to analyze the morphometry data (genotype x age). 

## Results           

### Baseline BP and HR in anesthetized mice

            To begin to determine whether BACHD mice show dysfunction in the cardiovascular system, we measured baseline BP and HR in lightly anesthetized WT (*n*=8) and BACHD mice (*n*=6; 7-9 mo of age).  Systolic BP was significantly higher in BACHD mice (108.0 ± 6.9 mmHg) compared to WT controls (86.1 ± 3.2 mmHg*; t_12_*=-3.13, *P*=0.009) although BP levels were still considered to be within the normal range.  BACHD mice also displayed increased HRs (615 ± 23 bpm) compared to WTs (537 ± 21 bpm; T=61.0, *P=*0.043).  The increased BP and HR measured in BACHD mice reflect a change in the regulation of the cardiovascular system.  Although the BP levels are still normal, the increase may be indicative of future susceptibility for cardiovascular events.

**

### Baroreceptor Reflex

            There is evidence that the ANS is dysfunctional in patients with Huntington’s disease.  It has also been reported that BACHD mice have significantly decreased HRV indicative of abnormal signal input to the heart by the ANS [16].  We measured the baroreceptor reflex of BACHD and WT mice to determine whether this feedback mechanism of regulating BP, which is mediated by the ANS, is also disrupted.  Changes in the ratios between the magnitude change in BP and HR after ATII (vasoconstrictor) or NP (vasodilator) administration, may indicate aberrant signaling to the heart by the ANS.    

            Increasing concentrations of ATII (LD-0.5, MD-1.0, HD-4.0 μg/kg) were administered to mildly anesthetized WT and BACHD mice. By 2-way repeated measures ANOVA, we detected significant variation between genotype (*F_1,55_*=7.28, *P*=0.019) and concentration of ATII drug (*F_3,55_*=16.94, *P*<0.001), but no significant interaction between genotype and drug concentration (*F_3,55_*=0.88, *P*=0.46; Fig. 1A).  Post *hoc* analysis indicate significant responses to all concentrations of AT II in WT mice when compared to the control conditions where the β- and muscarinic receptors were respectively blocked with propranolol and glycopyrrolate  before administration of a HD of ATII (control vs. LD: *t_14_=* 2.32, *P*<0.01; control vs. MD: *t_14_=* 2.13, *P*<0.001; control vs. HD: *t_14_=* 1.50, *P*<0.001).  Compared to controls, BACHD mice showed a significant difference in ∆HR/∆BP ratio when administered the LD and MD of ATII, but did not have a significant response to the HD (control vs. LD: *t_10_=* 3.49, *P*=0.008; control vs. MD: *t_10_=* 2.87, *P*=0.041; control vs. HD: *t_10_=* 2.31, *P*=0.16).  For each of the doses of ATII, the HR of BACHD mice responded with less magnitude compared to WT mice (LD: *t_12_=* 2.03, p=0.049; MD: *t_12_=* 2.21, p=0.033; HD: *t_12_=* 2.33, *P*=0.025).  There was no difference in the ∆HR/∆BP ratios between WT and BACHD mice when the β- and muscarinic receptors were blocked (control: *t_14_=* 0.58, *P*=0.56).

         To test the HR response of WT and BACHD mice to decreases in BP, we administered increasing concentrations of NP (LD- 10, MD- 20, HD- 40 μg/kg).  By 2-way repeated measures ANOVA, we detected significant interaction between genotype and concentrations of NP drug (*F_3,55_*=4.079, *P*=0.014, Fig. 1B).  Post *hoc* analysis detects a significant response to all concentrations of NP in WT mice when compared to the control (control vs. LD: *t_14_=* 5.62, *P*<0.001; control vs. MD: *t_14_=* 6.35, *P*<0.001; control vs. HD: *t_14_=* 6.89, *P*<0.001).  BACHD mice, on the other hand, did not show any significant response for any of the concentrations of NP when compared to the control condition (control vs. LD: *t_10_=* 1.43, *P*=0.96; control vs. MD: *t_10_=* 2.26, *P*=0.18; control vs. HD: *t_10_=* 1.66, *P*=0.64).  For each of the doses of NP, BACHD had significantly lower responses compared to WT mice (LD: *t_12_=* 3.32, *P*=0.002; MD: *t_12_=* 3.17, *P*=0.003; HD: *t_12_=* 4.05, *P*<0.001).  When mice were primed with β- and muscarinic receptor blockers, there was no difference in ∆HR/∆BP ratios between WT and BACHD mice after HD NP administration (control: *t_12_=* 0.46, *P*=0.65).

These data suggest that middle-aged BACHD mice have dysfunctional baro-receptor reflexes compared to WT controls.  BACHD mice are unable to appropriately modulate their HRs in response to either an increase or decrease in BP.  

**Figure:**
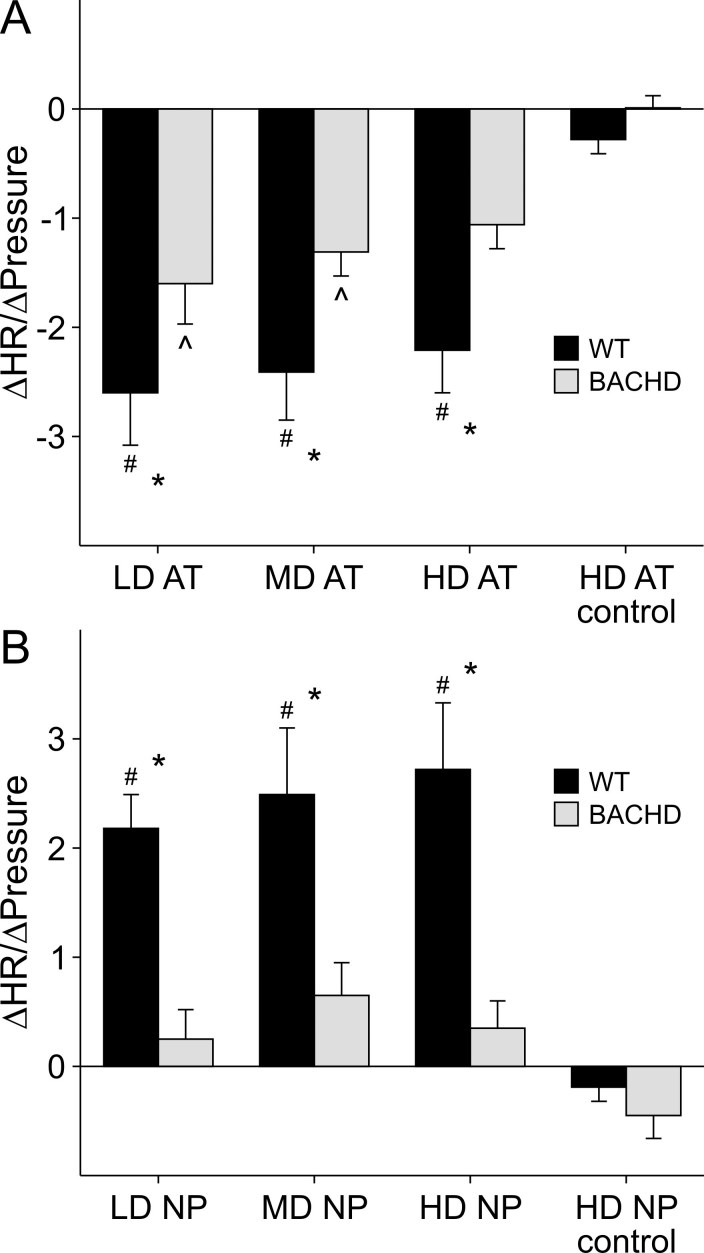
*Figure 1:* *Baroreceptor Reflex in WT and BACHD mice.* *Mice were administered increasing concentrations of ATII (A) and NP (B).* *For control conditions, mice were primed with muscarinic and* *β* *- blockers before administration of the HD of ATII and NP.* *The maximum change in HR (∆HR) and the absolute value of the maximum change in BP (∆BP) following drug administration was measured.* *We compared ∆HR/∆BP among the different groups using a 2-way repeated measures ANOVA.* *# significant response compared to control conditions in WT mice.* *^ significant response compared to control conditions in BACHD mice.* * significant difference between WT and BACHD mice. 

### Morphometry

            Increased BP and changes in baroreceptor reflex function may lead to cardiac hypertrophy.  We used a standard measure of heart morphometry by calculating the ratio of heart weight (HW) relative to body weight (BW) or tibia length (TL) in 7-8 mo and 12 mo old WT and BACHD mice.  

            In comparing the morphometry parameters among the various groups by 2-way ANOVA, we detected a significant effect of genotype on BW (*F_1,22_*=11.36, *P*=0.003), with BACHD mice displaying increased BW at 7mo. of age (*t_12_=3.31, P=*0.004) when compared to WT mice, but this difference is no longer detected between genotypes at 12 mo of age (*t_12_=1.49, P*=0.15; Table 1).  When comparing the HWs, we found a significant effect of genotype (*F_1,22_*=13.20, *P*=0.002) and age (*F_1,22_*=8.78, *P*=0.008), but no significant interaction between genotype and age (*F_1,22_*=0.13, *P*=0.72; Table 1).  The HWs of WT mice increased as they aged (*t_14_*=1.31, *P*=0.032), but at both age groups, the HW of BACHD mice were significantly higher when compared to WT mice (7mo.: *t*_12_ *=2.87, *P*=0.01; *12mo*:* *t_12_*=2.28, *P*=0.035).  Lastly, we found a significant effect of age (*F_1,22_*=7.16, *P*=0.015) when comparing TL (Table 1).  The TL of WT mice increased as they aged (*t_14_*=2.88, *P*=0.01), but this trend was not detected in BACHD mice.  Similar to BW, the TLs of BACHD mice were longer at 7mo. (*t_12_*= 2.43, *P*=0.025) compared to WT mice, but not at 12 mo of age (*t_12_*= 0.32, *P*=0.76). 

**Figure:**
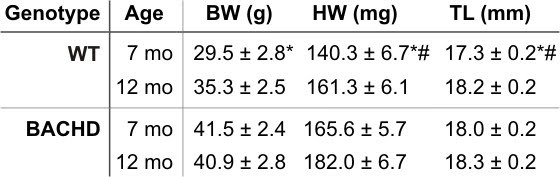
*Table 1:* Morphometry measurements of WT and BACHD mice. * significance between genotypes. *# significance between ages.*

            We did not find significant differences in the HW/BW ratios between WT and BACHD mice by 2-way ANOVA (age: *F_1,22_*=0.66, *P*=0.43; genotype: *F_1,22_*=2.85, *P*=0.108; age X genotype: *F_1,22_*=2.22, *P*=0.15), but measured significant differences in the HW/TL ratios between the two genotypes (Fig. 2).  By 2-way ANOVA, we find a significant effect of genotype (*F_1,22_*=10.50, *P*=0.004) and age (*F_1,22_*=5.31, *P*=0.033), but do not detect an interaction between genotype and age (*F_1,22_*=0.001, *P*=0.97).  The HW/TL ratios did not change as BACHD or WT mice aged (WT: *t_9_*=0.131, *P*>0.05; BACHD: *t_10_*=0.108, *P*>0.05), but the HW/TL ratios were significantly higher in BACHD mice compared to WT mice at both ages (7mo: *t_10_*=2.30, *P*=0.033; 12mo: *t_9_*=2.28, *P*=0.034).  These data indicate that the hearts of BACHD mice are increased in mass in relation to body frame and likely result in some form of cardiac remodeling. 

**

**Figure:**
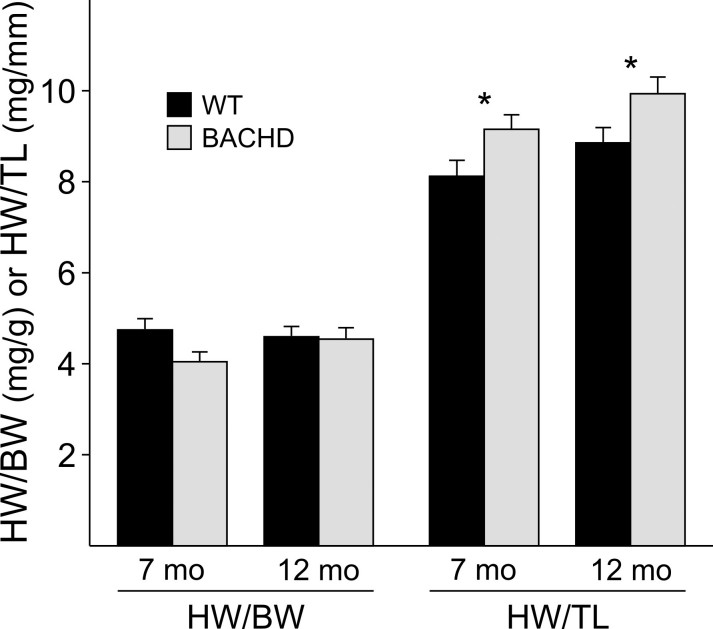
Figure 2:  Heart Morphometry comparisons between WT and BACHD mice at 7 mo and 12 mo of age.  HW/BW or HW/TL were calculated for all genotypes and age groups and compared using a 2-way ANOVA.                   * significant difference between genotypes.

### Echocardiographic Analysis

**Changes in the morphology of the hearts of BACHD mice, could cause aberrant modifications in heart function.  Therefore, we subjected BACHD and WT mice to echocardiographic analysis at about 12 mo of age in order to assess heart function.   We found no significant differences in any of the parameters measured (Table 2).   Though not statistically significant, the wall thicknesses and LV mass were slightly higher in the BACHD mice consistent with the morphometry data. Despite indications of abnormal regulation of BP and baroreflex, the hearts of BACHD mice continue to function normally at this age as assessed by echocardiogram.

**Figure:**
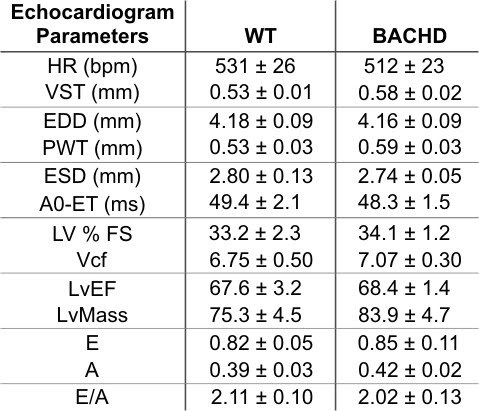
*Table 2: Echocardiogram parameters in 12 mo old WT and BACHD mice.* *No significant differences were detected between genotypes using a Student’s t-test.  HR- Heart Rate; VST-Ventricular Septal Thickness, EDD- End Diastolic Diameter, PWT- Posterior Wall Thickness, ESD- End Systolic Diameter, Ao-ET- Aortic Ejection Time, LV%FS- Left Ventricular percent Fractional Shortening, LvEF- Left Ventricular Ejection Fraction, LVMass- Left Ventricular Mass, and E/A- Early Diastole/Atrial contraction ratio.*

## Discussion

We report dysfunction of the ANS in BACHD mice, as measured by the baroreceptor reflex.  Furthermore, baseline measurements of BP and HR were higher in BACHD mice compared to WT controls.  Lastly, we find changes in the morphometry of the heart, but echocardiography did not detect any functional changes at 12 mo of age.   

The increased baseline BP levels measured in lightly anesthetized BACHD mice indicate changes in the overall regulation of BP tone.  Importantly, these BP recordings were taken during the day, a time in the resting period of mice when BP is expected to be low.  These increased BP levels parallel the reported increase in HR of BACHD mice also measured during the day [16], suggesting a global dysregulation of the cardiovascular system causing it to be hyperactive during the rest period.  The blunted depression in BP levels during rest would be similar to a nondipping pressure profile observed in humans, which in many cases is an indication of dysautonomia [22] [23].  Nondippers have a higher risk for future cardiovascular events, as well as damage to other organs [23] [24].  Published reports of BP in Huntington’s disease patients do not detect significant differences compared to control groups, however BP measurements were taken during the day when patients are in a more active state, which could mask the higher levels of BP tone [5] [7].  This appears to be the case for HR in BACHD mice, as HR levels are not significantly different between WT and BACHD mice during the active period [16].  Therefore, it may be valuable to examine nighttime BP and HR in Huntington’s disease patients, as well as pursue circadian studies of BP in awake and freely moving BACHD mice, which would further explore the extent of dysregulation in BP.  It is important to note that the BWs of BACHD mice are significantly increased at 7 mo of age compared to WT mice, possibly due to hypoactivity[16] [25], which may contribute to the observed increase in BP in BACHD mice.  This increased BW is transient, as the BWs are no longer significantly different at 12 mo of age.  Comparisons of BP at this older age may indicate whether heavier BW and/or other mechanisms of BP regulation are factors involved in the increase of BP observed in BACHD mice.

The BACHD mice showed deficits in the baroreceptor reflex, whereby the response in HR to the transient hyper- and hypotension induced by ATII and NP, respectively, is blunted compared to WTs.  This blunted response in both directions of the reflex suggests that both branches of the ANS may be affected.  The primary deficit in the baroreceptor reflex in BACHD mice is unknown, as this process is mediated by various regions of the brain that include the brainstem and hypothalamus, along with possible deficits in the outputs from the central autonomic nervous system [6] [18] [26].  *Htt* deposits are found globally in BACHD mice, which could disrupt function among cells in the baroreceptor pathway, even without evidence for gross degeneration of these brain regions [17].  In HD patients, the detection of alterations in the baroreceptor pathway, such as the vagal nuclei and cerebral cortex, are establishing structural cause for the dysfunction [5] [6] [27] [28] [29].  Ideally, identification of the site of dysfunction could be used to devise an appropriate therapeutic approach to manage symptomatic HD patients [30].  In humans, similar studies have been conducted to test the response of the baroreceptor reflex.  Results have been mixed, however recent studies report deficits during the Valsalva maneuver, hand-grip test, and the head up tilt test [4] [7] [31] [32].  Furthermore, patients complain of dizziness and light-headedness upon standing, which are symptoms of baroreceptor dysregulation resulting in orthostatic hypotension [6] [8].  Other measurements such as Heart Rate Variability and sympathetic skin response suggest that both branches of the ANS are disrupted in HD patients [4] [5] [6].  During the very early stages of HD, the sympathetic nervous system appears to be hyperactive [6] [7] [9].  As the disease advances, parasympathetic activity progressively decreases [4] [5] [7].  In our experiments, the blunted increase in HR after NP administration in BACHD mice may be a result of a ceiling effect for sympathetic activity, in that the range for further activation is limited due to an already hyperactive sympathetic nervous system.  The blunted decrease in HR after ATII administration may be a result of the dysfunction of both branches of the autonomic nervous system.  The hyperactivity of the sympathetic nervous system may not allow the HR to fully depress and in addition, the progressive dysfunction of the parasympathetic nervous system may be inadequate to slow HR.  Tests to measure both the sympathetic and parasympathetic nervous system activity in BACHD mice would be able to determine the extent of the dysfunction of each of the branches.

There is a high prevalence of autonomic dysfunction in Huntington’s disease patients that manifest as various clinical symptoms and signs such as gastrointestinal complaints, urinary difficulties and postural dizziness [8].  More profound may be the increased risk of cardiac arrhythmias, and the development of coronary heart disease, that result in a significant number of deaths within the Huntington’s disease population [2] [3] [5] [33].  Importantly, these autonomic symptoms occur before any motor deficits, therefore measures of autonomic dysfunction could be a valuable tool in Huntington’s disease diagnosis and staging [8] [9].  Upon diagnosis, vigilant monitoring of the cardiovascular system may help deter the incidence of fatal events.  We were able to measure changes in the morphometry of the heart in BACHD mice relative to body frame size (TL), which could indicate the start of cardiac remodeling in response to the increased BP and aberrant autonomic signaling.  Echocardiogram results do not show functional changes in the hearts of BACHD mice, suggesting that the hearts at this stage of the disease are still able to compensate despite the morphological and physiological changes in the cardiovascular system.  The R6/2 Huntington disease mouse model, which display a more rapid disease progression, develop serious cardiac dysfunction suggesting a cardiotoxic effect of *htt *deposits in the heart [15], and we speculate that BACHD mice may develop cardiac dysfunction at an older age.  Future work can be directed to study the susceptibility of this BACHD model to cardiovascular stresses as well as design management strategies focused on cardiovascular health to offset and reduce the chances of serious cardiovascular events in HD patients. 

## Funding information

Work supported by NRSA award F31NS070529 to AMS as well as CHDI Foundation Grant A-4339 and a Stein Oppenheimer award to CSC.

## Competing interests

The authors have declared that no competing interests exist.
